# Characterization of deep-sea benthic invertebrate megafauna of the Galapagos Islands

**DOI:** 10.1038/s41598-020-70744-1

**Published:** 2020-08-17

**Authors:** Pelayo Salinas-de-León, Patricia Martí-Puig, Salome Buglass, Camila Arnés-Urgellés, Etienne Rastoin-Laplane, Marie Creemers, Stephen Cairns, Charles Fisher, Timothy O’Hara, Bruce Ott, Nicole A. Raineault, Henry Reiswig, Greg Rouse, Sonia Rowley, Timothy M. Shank, Jenifer Suarez, Les Watling, Mary K. Wicksten, Leigh Marsh

**Affiliations:** 1 Charles Darwin Research Station, Charles Darwin Foundation, Av. Charles Darwin s/n, Puerto Ayora, Santa Cruz, Galapagos Islands, Ecuador; 2 Pristine Seas, National Geographic Society, Washington, DC USA; 3 National Museum of Natural History, W-205, MRC 163, Smithsonian, 10th & Constitution, Washington, DC USA; 4 Department of Biology, Pennsylvania State University, University Park, PA 16802 USA; 5 Museum Victoria, Melbourne, VIC 3053 Australia; 6 Khoyatan Marine Laboratory, North Saanich, BC, Canada; 7 Ocean Exploration Trust, Narragansett, Rhode Island USA; 8 University of Victoria and Royal B.C. Museum, Victoria, BC Canada; 9 Scripps Institution of Oceanography, La Jolla, CA 92037 USA; 10 Department of Earth Sciences, University of Hawaii At Manoa, Honolulu, HI USA; 11 Woods Hole Oceanographic Institution, Woods Hole, MA 02543 USA; 12 Dirección del Parque Nacional Galápagos, Av. Charles Darwin s/n, Puerto Ayora, Galápagos Islands Ecuador; 13 Department of Biology, University of Hawaii At Manoa, Honolulu, HI USA; 14 Department of Biology, Texas A&M University, College Station, Texas 77843-3258 USA; 15 Ocean and Earth Science, University of Southampton, Waterfront Campus, Southampton, SO14 3ZH UK

**Keywords:** Biodiversity, Community ecology

## Abstract

The deep sea represents the largest and least explored biome on the planet. Despite the iconic status of the Galapagos Islands and being considered one of the most pristine locations on earth, the deep-sea benthic ecosystems of the archipelago are virtually unexplored in comparison to their shallow-water counterparts. In 2015, we embarked on a multi-disciplinary scientific expedition to conduct the first systematic characterization of deep-sea benthic invertebrate communities of the Galapagos, across a range of habitats. We explored seven sites to depths of over 3,300 m using a two-part Remotely Operated Vehicle (ROV) system aboard the E/V *Nautilus,* and collected 90 biological specimens that were preserved and sent to experts around the world for analysis. Of those, 30 taxa were determined to be undescribed and new to science, including members of five new genera (2 sponges and 3 cnidarians). We also systematically analysed image frame grabs from over 85 h of ROV footage to investigate patterns of species diversity and document the presence of a range of underwater communities between depths of 290 and 3,373 m, including cold-water coral communities, extensive glass sponge and octocoral gardens, and soft-sediment faunal communities. This characterization of Galapagos deep-sea benthic invertebrate megafauna across a range of ecosystems represents a first step to study future changes that may result from anthropogenic impacts to the planet’s climate and oceans, and informed the creation of fully protected deep-water areas in the Galapagos Marine Reserve that may help preserve these unique communities in our changing planet.

## Introduction

When Charles Darwin visited the Galapagos Islands aboard the HMS Beagle in 1835^1^, deep-sea exploration was in its infancy and the presence of life at depth was questioned^2,3^. The Galapagos served as inspiration for Darwin’s breakthrough theory of evolution by means of natural selection^4^ and ever since, the iconic islands have captivated the global scientific community. While the Galapagos terrestrial fauna and flora have been the subject of intense study^5–7^, and research on shallow (< 40 m) coastal marine ecosystems has been considerable over the past four decades^8–12^, only a limited number of expeditions have explored beyond SCUBA diving depths. Most of these deep-sea research efforts have followed on the historic discovery of hydrothermal vents on the Galapagos Spreading Centre^13,14^ and further investigations on the volcanic origin of the islands^15,16^. A limited number of expeditions have been dedicated to the study of deep biological communities, with the 1986 Johnson Sealink being the most prolific in terms of invertebrate collections. However, the 25-year sequestration period placed by the National Cancer Institute on all specimens collected has limited the number of scientific publications from this cruise until recently^24^. The other limited number of expeditions to the Galapagos have been focused on the chemolithoautotrophic fauna associated with hydrothermal vents^17–19^, specific taxa such as fish^20–23^ and deep-sea corals^24,25^, or investigating the invertebrate communities associated with soft-sediment environments by means of trawling^26^.

Despite our fascination for the earth’s final frontier, the proportion of the deep sea that has been explored and sampled is minimal across all ocean basins^27^. Recent technological advances have facilitated deep-sea exploration to unprecedented levels, resulting in the discovery of new and diverse ecosystems^27^, the description of thousands of species new to science^28^ and the documentation of unique behaviours and natural histories^29–31^. With an estimated one-third to two-thirds of species in the ocean still awaiting description^28^, the deep sea has the potential to host a large proportion of earth’s undiscovered biodiversity. As our footprint on earth keeps growing and human activities now threaten the deeper parts of our oceans, it is key that we document and inform management actions to protect the biodiversity of the deep sea before it is too late.

The volcanic and isolated nature of the Galapagos Islands, represents an oasis of life in the vastness of the Eastern Pacific abyssal plain^32^, with the presence of numerous seamounts around the archipelago^33^. Among deep-sea environments, seamounts are known to support some of the most diverse and productive habitats, as physical interactions at these topographically pronounced features and surrounding water masses create favourable conditions that enhance biodiversity, species richness and possible endemism^34–37^. The location of the Galapagos Islands, at the crossroads of warm and cold oceanic currents, also results in a unique oceanographic setting^38^, with three major biogeographical groupings reported for shallow-water reef fauna^39^. Whether this is reflected on deep-sea taxa is unknown. Moreover, destructive human practices such as bottom trawling, globally known to have catastrophic impacts on slow-growing sessile deep-sea communities^40,41^, have been historically absent across the archipelago. As a result, the Galapagos Islands are likely to harbour unique, pristine deep-sea invertebrate benthic communities. In 2015, we explored the deep-water regions of the Galapagos Islands using a two-part Remotely Operated Vehicle (ROV) system aboard the E/V *Nautilus.* Here we present the results of a systematic characterization of deep-sea benthic invertebrate communities of the Galapagos across a range of habitats and investigate biodiversity across the different locations and depths explored.

## Results and discussion

**Figure 1:**
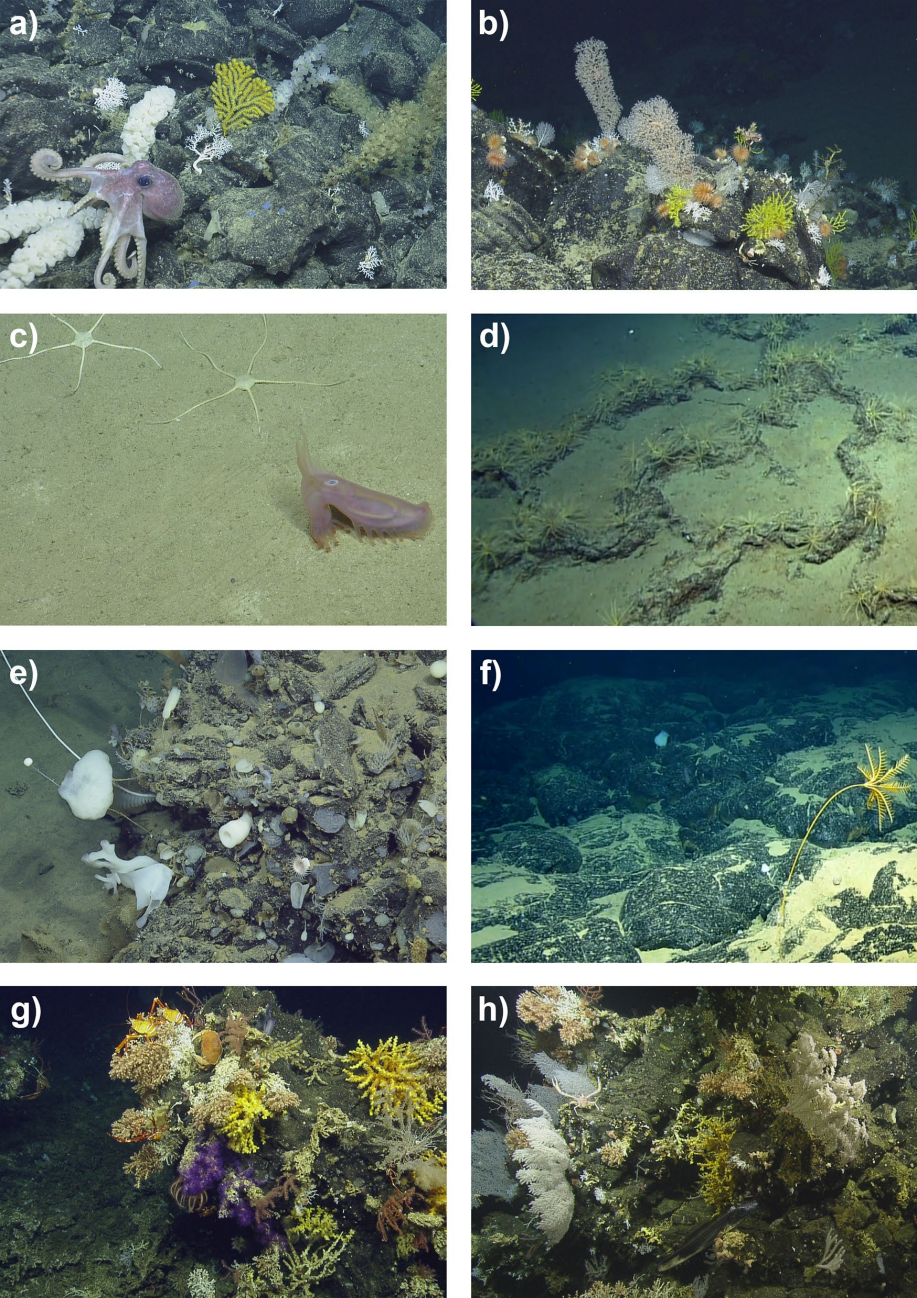
Examples of the habitats and taxa present on the seamounts and lava flows of the Galapagos Marine Reserve (GMR) observed during the NA064 EV *Nautilus* expedition. (**a**) Dive H1435 seamount, depth 1,014 m. Octocorals and Hexactinellida sponges associated with volcanic substrate; (**b**) Dive H1435 seamount, depth 813 m. Scleractinia and Octocorallia associated with volcanic substrate; (**c**) Dive H1436 seamount, depth 2086 m. Ophiuroids and holothurian *Peniagone* indet. associated with an area of sand; (**d**) Dive H1440 seamount, depth 1,427 m. Aggregation of comatulid crinoids on exposed volcanic substrate; (**e**) Dive H1441 lava flow, depth 3,388 m. A mixed sponge and octocoral community on volcanic outcrop; (**f**) Dive H1442 lava flow, depth 2,922 m. A stalked sponge and *Bathycrinus* sp. crinoid on lava pillows; (**g**) Dive H1443 seamount, depth 414 m. Squat lobsters and brachyuran crabs on Scleractinia and Octocorallia colonies; (**h**) Dive H1443 seamount, depth 446 m. A mixed community of Scleractinia and Octocorallia colonies. *Note; not all taxa presented in this figure were collected for taxonomic identification.*

Throughout the ROV surveys we documented the presence of a range of underwater communities between depths of 290 and 3,373 m, including cold-water coral communities, extensive glass sponge and octocoral gardens, and soft-sediment faunal communities (Fig. 1; Table 1). A total of 90 taxa from six phyla, 13 classes, 22 orders and 44 families were collected and identified to the lowest taxonomic level possible by experts. Of the specimens analysed, thirty were identified as species new to science. Eleven of these taxa were also identified as belonging to new undescribed genera (Table 2).

**Table 1:** Summary of the ROV dive transects, sampling events, presence of vulnerable marine ecosystems and environmental parameters within the Galapagos Marine Reserve (GMR) during expedition NA064.

Dive	H1435	H1436	H1440	H1441	H1442	H1443A	H1443B
Duration	16:48:47	11:14:19	11:23:43	13:34:44	18:22:52	10:02:24	06:39:17
Feature	Seamount	Seamount	Seamount	Abyssal Plain	Abyssal Plain	Seamount	Seamount
Latitude	1.2105	1.6584	1.8535	− 0.3763	− 0.3763	− 0.3824	− 0.3824
Longitude	− 91.0836	− 91.6731	− 92.1064	− 91.9043	− 91.7619	− 90.8091	− 90.8091
Geographic location	East of Wolf	East of Darwin	North of Darwin	West of Fernandina	West of Fernandina	West of Santiago	West of Santiago
Transect length (m)	5,980	5,947	6,958	9,551	9,184	3,208	3,882
Number of sampling events^†^	19 (15)	15 (8)	18 (5)	16 (3)	25 (6)	18 (5)	5 (0)
Total number of species identified	48	20	5	3	6	6	0
Undescribed/new to science*	16 (31%)	5 (25%)	3 (60%)	2 (67%)	1 (17%)	3 (50%)	0 (NA)
Vulnerable Marine Ecosystems (VMEs)	Cold-water corals Coral gardens Sponge gardens	*Indicator species present	*Indicator species present	*Indicator species present	*Indicator species present	Cold-water corals Coral gardens Sponge gardens	Cold-water corals Coral gardens
Depth range (m)	290–1,199	930–2088	1,188–1962	3,307–3,373	2,940–3,012	411–640	248–618
Temperature range (°C)	3.88–13.45	2.28–4.96	2.54–3.84	1.79	1.76	7.15–9.10	7.15–13.61
Salinity range (PSU)	33.64–35.04	34.56–34.66	34.34–34.66	34.67	34.68	34.54–34.74	34.6–35.00
O_2_ range (µmol/L)	6.87–79.94	67.56–115.52	80.52–106.69	140.48–140.78	141.80–141.93	27.03–40.00	15.42–66.85

(^†^)value in brackets represents number of biological samples collected (*) value in brackets represents percentage of biological samples that are either undescribed or new to science from each dive location.

(^+^)Indicator species are those listed in the VME criteria under the methods section (octocoral, hexacorals, scleractinians, and erect sponges) but do not meet the required abundances.

**Table 2:** List of deep-sea invertebrate megafauna sampled during the NA064 Galapagos cruise aboard the E/V Nautilus. New species are in bold.

Sample ID	Event	Dive	Depth (m)	Phylum	Class	Order	Family	Lowest taxonomic ID	Authority	ID
NA064-028–01-03-A	028	H1436	1,199	Annelida	Polychaeta	Phyllodocida	Polynoidae	*Macellicephala galapagensis*	(McIntosh, 1885)	GR^†^
NA064-027–01-01-A	027	H1436	1,272					*Melaenis* indet.		GR^†^
NA064-019–03-A	019	H1435	296					*Hyperhalosydna* indet.		GR^†^
NA064-034–01-02-A	034	H1436	973					*Macellicephala* indet. sp. 1		GR^†^
NA064-004–08-A	004	H1435	1,153					*Macellicephala* indet. sp. 2		GR^†^
NA064-029–05-A	029	H1436	1,047					*Macellicephala* indet. sp. 3		GR^†^
NA064-018–01-01-A	018	H1435	299					Polynoinae indet. sp. 1		GR^†^
NA064-014–01-09-A	014	H1435	871					Polynoinae indet. sp. 2		GR^†^
NA064-011–01-03-A	011	H1435	1,052	Arthropoda	Malacostraca	Decapoda	Axiidae	*Eiconaxius albatrossae*	Kensley, 1996	MW
NA064-013–02-01-A	013	H1435	873				Bathypalaemonellidae	*Bathypalaemonella cf. serratipalma*	Pequegnat, 1970	LW
NA064-013–03-01-A	013	H1435	873				Chirostylidae	*Sternostylus defensus*	(Benedict, 1902)	MW
NA064-010–01-02-A	010	H1435	1,067					***Uroptychus compressus*** **sp. nov.**	Baba & Wicksten, 2019	MW
NA064-007–01-03-B	007	H1435	1,041					*Uroptychus* indet		MW
NA064-013–01-01-A	013	H1435	873					*Uroptychus occidentalis*	Faxon, 1893	MW
NA064-130–01-01-A	130	H1443	472					*Uroptychus bellus*	Faxon, 1893	MW
NA064-031–01-01	031	H1436	1,012					***Heteroptychus galapagos*** **sp. nov.**	Baba & Wicksten, 2019	MW
NA064-009–01-01-A	009	H1435	1,048					***Heteroptychus nautilus*** **sp. nov.**	Baba & Wicksten, 2019	MW
NA064-018–01-03-A	018	H1435	305				Eumunididae	***Eumunida subsolanus*** **sp. nov.**	Baba & Wicksten, 2019	MW
NA064-019–02-B	019	H1435	296				Mithracidae	*Mithraculus* indet.		TS^†^
NA064-004–03-A	004	H1435	1,153				Munididae	*Munida perlata*	Benedict, 1902	MW
NA064-004–01-A	004	H1435	1,153				Munidopsidae	*Munidopsis* indet.		MW
NA064-028–01-04-A	028	H1436	1,199					*Munidopsis mina*	Benedict, 1902	MW
NA064-027–01-03-A	027	H1436	1,272					*Munidopsis modesta*	Benedict, 1902	MW
NA064-122–02-A	122	H1443	464					*Munidopsis scabra*	Faxon, 1893	MW
NA064-022–01-01-A	022	H1436	1607				Nematocarcinidae	*Nematocarcinus* indet		TS^†^
NA064-130–01-02-A	130	H1443	472				Palaemonidae	*Pontonides sympathes*	(De Ridder & Holthuis, 1979)	MW
NA064-019–15-A	019	H1435	296		Pycnogonida	Pantopoda	Ammotheidae	Ammotheidae indet.	Dohrn, 1881	MW
NA064-067–01-A	067	H1440	1,222	Cnidaria	Anthozoa	Alcyonacea	Acanthogorgiidae	*Acanthogorgia* indet. sp. 1		SR
NA064-005–01-A	005	H1435	1,153					*Acanthogorgia* indet. sp. 2*		SR
NA064-007–02-A	007	H1435	1,041					*Acanthogorgia* indet. sp. 3		SR
NA064-026–01-A	026	H1436	1,286					*Acanthogorgia* indet. sp. 4		SR
NA064-076–01-A	076	H1441	3,379				Alcyoniidae	***Bathyalcyon*** sp. nov.*		LW
NA064-013–01-A	013	H1435	873				Chrysogorgiidae	*Chrysogorgia* indet.		LW
NA064-079–01-A	079	H1441	3,382				Isididae	***Bathygorgia*** **sp. nov.**		LW
NA064-012-A	012	H1435	1,046					**Isididae gen. nov. (clade H1)**		LW
NA064-121–01-A	121	H1443	464					**Isididae gen. nov. sp. 1**		LW
NA064-055–01-A	055	H1440	1822					**Isididae gen. nov. sp. 2**		LW
NA064-061–01-A	061	H1440	1,407					Isididae gen. nov. sp. 3*		LW
NA064-099–01-A	099	H1442	2,923					**Isididae gen. nov. sp. 4**		LW
NA064-009–01-A	009	H1435	1,048					**Isididae gen. nov. (clade 1) sp. 1**		LW
NA064-021–01-A	021	H1436	1634					**Isididae gen. nov. (clade 1) sp. 2**		LW
NA064-031–01-A	031	H1436	1,011					**Isididae gen. nov. (clade 1) sp. 3**		LW
NA064-062–01-A	062	H1440	1,405					**Isididae gen. nov. (clade 1) sp. 4**		LW
NA064-019–01-A	019	H1435	296				Plexauridae	*Swiftia* indet.sp. 1		SR
NA064-007–01-A	007	H1435	1,041					*Swiftia* indet.sp. 2		SR
NA064-077–01-A	077	H1441	3,381				Primnoidae	*Callozostron carlottae*	Kükenthal, 1909	SC
NA064-126–01-A	126	H1443	445					***Calyptrophora*** **sp. nov.**		SC
NA064-125–01-A	125	H1443	446					***Parastenella*** **sp. nov.**		SC
NA064-034–01-A	034	H1436	973				Victorgorgiidae	***Victorgorgia*** sp. nov.*		LW
NA064-018–01-10-A	018	H1435	299	Mollusca	Gastropoda	Lepetellida	Fissurellidae	Fissurellidae indet.	J. Fleming, 1822	GR^†^
NA064-107–01-A	107	H1442	2,919	Echinodermata	Asteroidea	Brisingida	Brisingidae	*Hymenodiscus pannychia*	(Fisher, 1928)	GR^†^
NA064-003–01-A	003	H1435	1,178			Valvatida	Solasteridae	Solasteridae indet.		GR^†^
NA064-027–01-02-A	027	H1436	1,272		Crinoidea	Comatulida	Antedonidae	*Fariometra* indet.		GR^†^
NA064-071–06-A	071	H1440	1,338					*Fariometra* cf. parvula	(Hartlaub, 1895)	GR^†^
NA064-011–01-01-A	011	H1435	1,052					Heliometrinae indet.		GR^†^
NA064-097–01-A	097	H1442	2,949				Bathycrinidae	*Bathycrinus cf. equatorialis*	AH Clark, 1908	GR^†^
NA064-022–01-A	022	H1436	1607				Pentametrocrinidae	*Pentametrocrinus paucispinulus*	Messing, 2008	GR^†^
NA064-029–02-A	029	H1436	1,047				Thalassometridae	Thalassometridae indet. sp. 1		GR^†^
NA064-096–01-A	096	H1442	2,922					Thalassometridae indet. sp. 2		GR^†^
NA064-028–01-A	028	H1436	1,199		Echinoidea	Aspidodiadematoida	Aspidodiadematidae	*Plesiodiadema* indet. sp. 1		GR^†^
NA064-103–01-A	103	H1442	2,908					*Plesiodiadema* indet. sp. 2		GR^†^
NA064-017–11-A	017	H1435	352			Cidaroida	Cidaridae	Cidaridae indet. sp. 1		GR^†^
NA064-018–01-11-A	018	H1435	299					Cidaridae indet. sp. 2		GR^†^
NA064-105–01-A	105	H1442	2,914		Holothuroidea	Synallactida	Deimatidae	*Oneirophanta setigera*	(Ludwig, 1893)	GR^†^
NA064-004–07-A	004	H1435	1,153		Ophiuroidea	Amphilepidida	Ophiothamnidae	*Histampica duplicata*	(Lyman, 1875)	TO
NA064-026–01-01-A	026	H1436	1,286			Euryalida	Euryalidae	*Asteroschema* indet. sp. 1		TO
NA064-005–01-01-A	005	H1435	1,153					*Ophiocreas* indet. sp. 1		TO
NA064-017–03-A	017	H1435	352			Ophiacanthida	Ophiacanthidae	*Ophiacantha contigua*	Lütken & Mortensen, 1899	TO
NA064-015–05-A	015	H1435	376					*Ophiacantha quadrispina*	H.L. Clark, 1917	TO
NA064-007–01-02-A	007	H1435	1,041					*Ophiacantha similis*	A.H. Clark, 1916	TO
NA064-027–01-05-A	027	H1436	1,272					*Ophiolebes mortenseni*	A.H. Clark, 1916	TO
NA064-015–13-A	015	H1435	376					*Ophiolimna bairdi*	Lyman, 1883	TO
NA064-027–01-04-A	027	H1436	1,272					*Ophioplinthaca* sp. nov.		TO
NA064-015–06-A	015	H1435	376					*Ophiotreta valenciennesi*	(Lyman, 1879)	TO
NA064-004–06-A	004	H1435	1,153			Ophiurida	Ophiuridae	***Ophiocten*** **sp. nov.**		TO
NA064-004–10-A (a)	004	H1435	1,153	Porifera	Demospongiae	Desmacellida	Desmacellidae	***Desmacella*** **sp. nov.**		BO
NA064-015–07-A	015	H1435	376			Haplosclerida	Chalinidae	***Haliclona*** **sp. nov.**		BO
NA064-004–10-A (b)	004	H1435	1,153					*Haliclona (Gellius) cf. perforata*	Wilson, 1904	HR
NA064-029–04-A	029	H1436	1,047				Niphatidae	***Pachychalina*** **sp. nov.**		BO
NA064-029–01-A	029	H1436	1,047			Poecilosclerida	Cladorhizidae	*Asbestopluma* indet.		HR
NA064-010–01-A	010	H1435	1,067				Phellodermidae	Phellodermidae gen. nov.*		HR
NA064-018–01-08-A (c)	018	H1435	299					Phellodermidae indet.*		BO
NA064-019–10-A	019	H1435	296			Polymastiida	Polymastiidae	***Polymastia*** sp. nov.*		BO
NA064-018–01-08-A (a)	018	H1435	299			Suberitida	Halichondriidae	***Hymeniacidon*** sp. nov.*		BO
NA064-019–14-A	019	H1435	296				Suberitidae	*Protosuberites*sp. nov.*		BO
NA064-019–08-A	019	H1435	296					***Prosuberites*** sp. nov.*		BO
NA064-018–01-08-A (b)	018	H1435	299		Hexactinellida	Lyssacinosida	Rossellidae	***Vitrollula*** sp. nov.*		BO
NA064-011–01-A	011	H1435	1,052			Sceptrulophora	Euretidae	*Conorete erectum*	(Schulze, 1899)	HR
NA064-006-A	006	H1435	1,048				Farreidae	Farreidae gen. nov.*		HR
NA064-014–01-A	014	H1435	871				Tretodictyidae	***Tretodictyum*** **sp. nov.**		HR

*GR* Greg Rouse, *MW* Mary K. Wicksten, *TS* Tim Shank, *SR* Sonia Rowley, *LW* Les Watling, *SC* Stephen Cairns, *TO* Tim O’Hara, *BO* Bruce Ott, *HR* Henry Reiswig.

**Figure 2:**
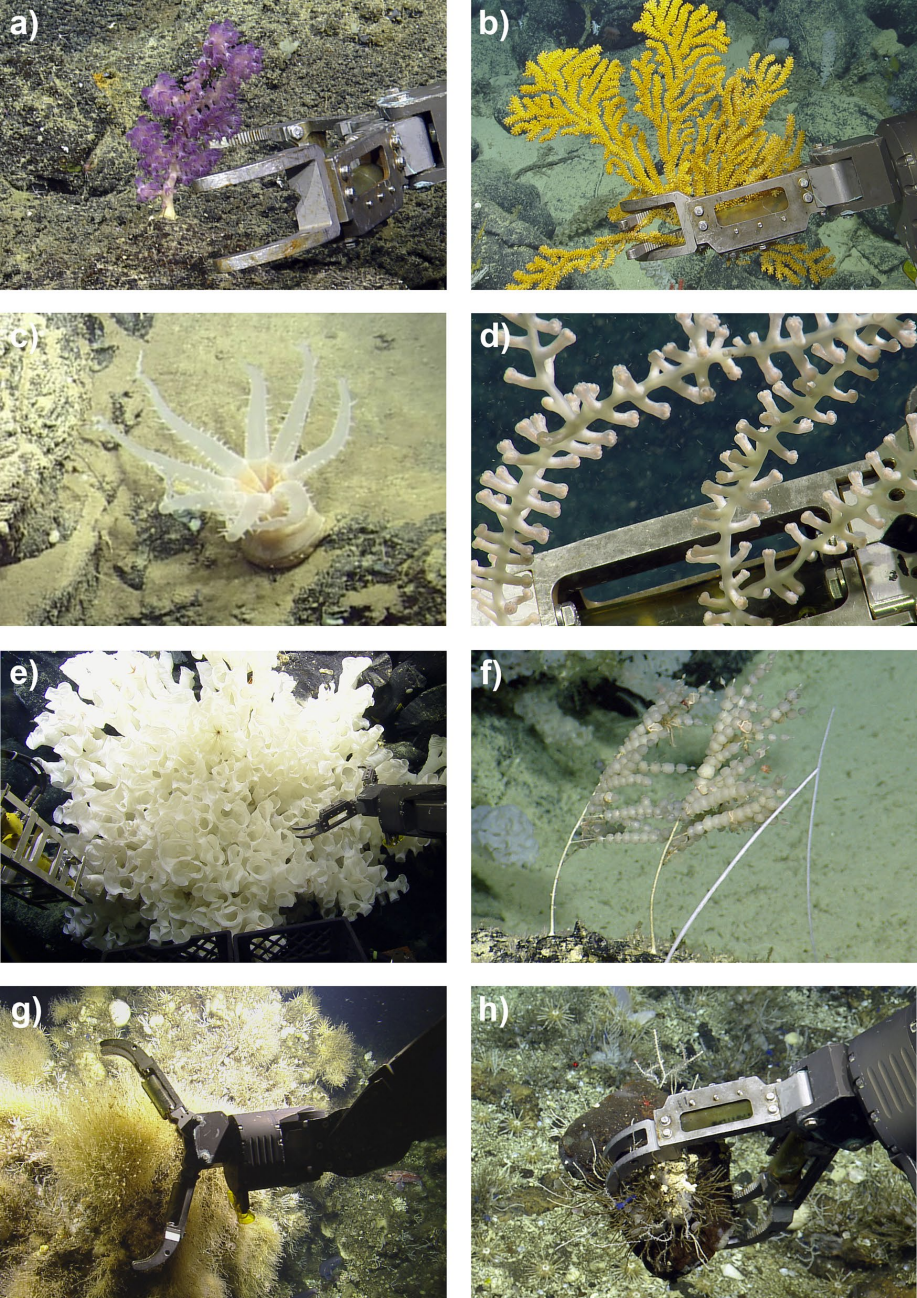
Examples of voucher specimens collected and identified from the NA064 EV *Nautilus* expedition (**a**) Dive H1436, depth 974 m. NA064-034 *Victorgorgia* sp. nov.; (**b**) Dive H1435, depth 1,042 m. NA064-007 *Acanthogorgia* indet. sp 2; (**c**) Dive H1441, depth 3,382 m. NA064-076 *Bathyalcyon* sp. nov.; (**d**) Dive H1440, depth 1,404 m. NA064-061 Isididae gen. nov.; (**e**) Dive H1435, depth 1,048 m. NA064-006 Farreidae gen. nov.; (**f**) Dive H1435, depth 1,068 m. NA064-010 Phellodermidae gen. nov.; (**g**) Dive H1435, depth 305 m. NA064-018 Mixed sponge colony. Specimens examined within this sample have been identified to 3 different taxa. *Vitrollula* sp. nov; *Hymeniacidon* sp. nov; Phellodermidae indet.; (**h**) Dive H1435, depth 297 m. NA064-019 Various sponges. All specimens identified to genus: *Polymastia* sp. nov; *Protosuberites* sp. nov.; *Prosuberites* sp. nov.

*Indicates voucher specimen shown in Fig. 2. ^†^Indicates specimens that were sequenced. All other identified from morphology.

Some of the new taxa discovered includes a giant solitary coral of the genus *Bathyalcyon* (Fig. 2c), the first known for the region; a new genus of bamboo corals of the family Isididae, with this specimen recorded at 2,923 m deep (Fig. 2d); a new genus of glass sponges of the family Farreidae, with some of the colonies encountered growing over 1 m in width (Fig. 2e); and a new demosponge genus in the family Phellodermidae, with an unusual ‘kebab-like’ growth form (Fig. 2f).

This study represents one of the most comprehensive surveys of deep-sea invertebrate megafauna across a varied range of habitats, depth gradients and geographical locations within the Galapagos Islands. To our knowledge, this study provides the first systematic description of deep-sea specimens of several phyla, including Porifera, Echinodermata, Arthropoda and Annelida for Galapagos and also the Tropical Eastern Pacific (TEP)^42^. We also provide additional deep-water octocoral (Anthozoa) discoveries, a group that has previously received by far the most scientific attention from deep Galapagos waters^24,25,42^. Heterogeneous environments were found on many of the seamounts explored, including the presence of multiple fragile habitats, such as glass sponge gardens, coral gardens and cold-water coral colonies, that are considered Vulnerable Marine Ecosystems (VMEs) by the United Nations General Assembly^43,44^.

Our analysis of ROV imagery, revealed that VME indicator species were present on all the dives conducted, although their presence varied considerably among locations. According to the criteria used in our image analysis, VME communities were present on three of the sites visited, including one located on the far north bioregion of the archipelago (Dive H1435) and two sites within the central-south-eastern bioregion (Dive H1443; Table 3). The presence of cold-water corals was restricted to depths shallower than 500 m, while coral and sponge gardens, dominated by hexactinellids, were documented to depths of around 1,000 m (Table 3). The limited presence of VME communities on the remaining four sites sampled (Dives H1436; H1440; H1441; H1442) might be explained in part by the differences in the depths sampled, with ROV transects at these sites conducted at depths greater than 930 m, and the structural complexity of the seafloor. Dives H1436 and H1440, were conducted at seamounts with a much deeper, less topographically complex slope to the summit, while dives H1441 and H1442, were conducted on a predominantly sediment-covered seafloor, and only experienced a depth change of 66 m and 72 m respectively (Supplementary material I).

**Table 3:** Summary of the vulnerable marine ecosystems present within the Galapagos Marine Reserve (GMR) during expedition NA064.

Dive	Cold-water corals	Coral gardens	Sponge gardens	Total % of VMEs in images analysed
**H1435**				
Depth range present	359–449 m	293–1102 m	293–1165 m	42%
Percentage presence over dive transect	5%	19%	33%	
**H1443A**				
Depth range present	414–489 m	417–618 m	417–618 m	27%
Percentage presence over dive transect	10%	37%	3%	
**H1443B**				
Depth range present	249–335 m	252–514 m	Not Present	46%
Percentage presence over dive transect	4%	14%	Not Present	

**Figure 3:**
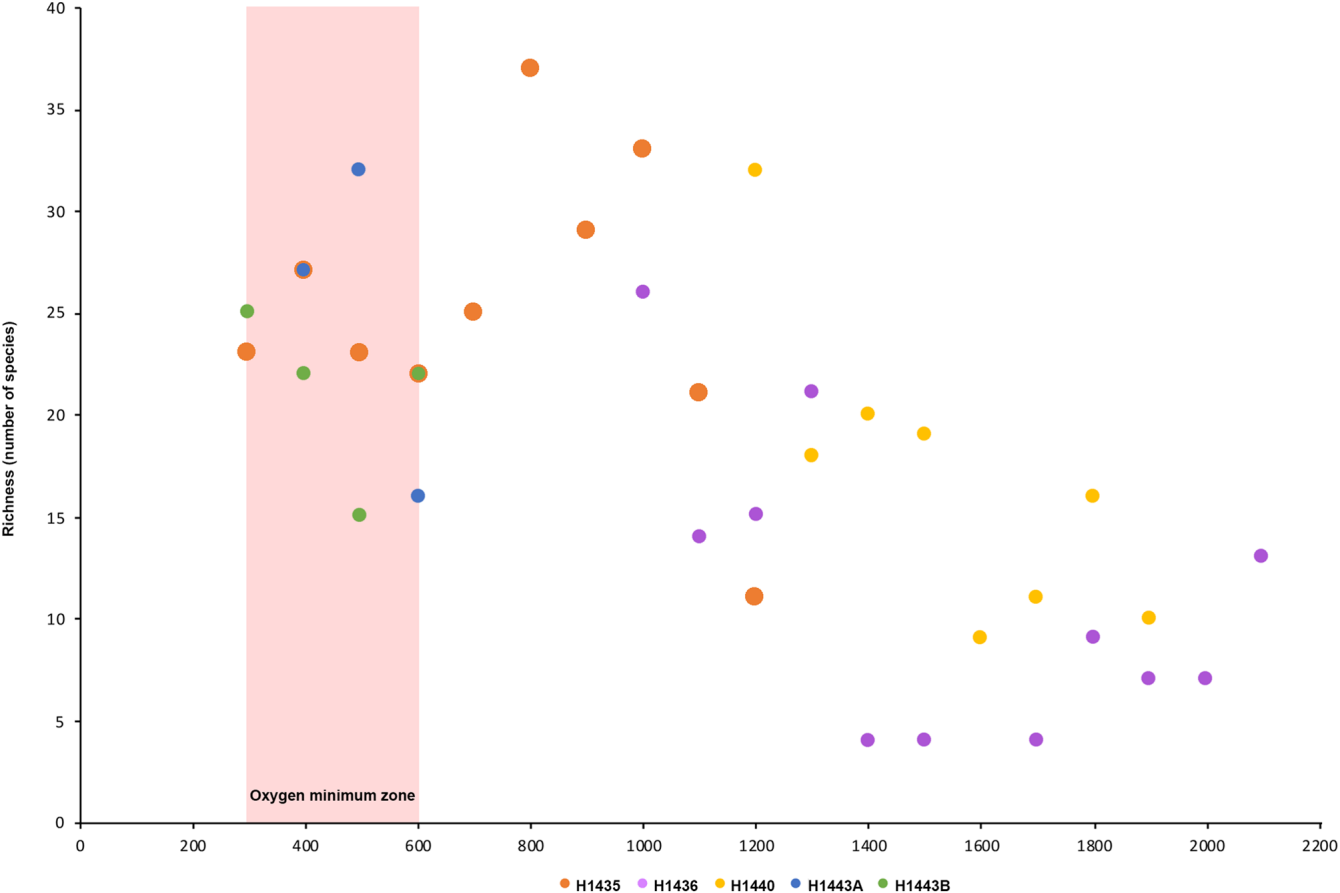
Depth-related trends in morphospecies richness per 100 m-depth band reported on three seamounts (H1435, H1436, H1440) and two volcanic cones (H1443A, H1443B) of the Galapagos Marine Reserve. The distribution of the Oxygen Minimum Zone (OMZ) as recorded on the ROV mounted CTD and Oxygen optode (Supplementary Material III) is highlighted in red.

Our systematic analysis of ROV imagery documented a total of 70 benthic megafauna morphospecies (or morphospecies groups) across the locations surveyed (Supplementary Material II). The most speciose phyla observed were cnidarians (n = 31), echinoderms (n = 14), and arthropods (n = 9). Morphospecies of sponges, molluscs, annelida and tunicates were also observed (Fig. 1). In order to compare richness across dive locations and depth, morphospecies richness was derived from the number of the species present in each 100 m depth band. Analyses revealed that Dive H1435 in the Far north region of the GMR displayed the highest values in species richness within the 700–1,400 m depth strata, with a peak of 37 different morphospecies recorded at 800 m (Fig. 3). The lowest species richness was recorded at the deeper locations, with a minimum richness of four morphospecies recorded at 1,400–1,700 m on Dive H1436, also in the far north region of the GMR (Fig. 3).

The detailed species inventory from physical samples and spatial and depth related biodiversity patterns presented here provides one of the first comprehensive characterizations within the TEP, a marine ecoregion of South America where benthic marine biodiversity is presently poorly known and underestimated^45^. It also contributes to the global effort to publish deep-sea biodiversity inventories, a step critical to identify VME indicator species and communities in an effort to develop long-term conservation management plans for these areas on both national and regional scales. To reach these goals, there is a need to expand the taxonomic coverage of collections and compile this information from regional and global datasets^46^. In this study, we had the support and collaboration of taxonomic experts for each phylum from all over the world; nevertheless, taxonomy is still a major constraint on such studies as there is a general shortage of taxonomic expertise for marine biodiversity, particularly for the TEP^45^.

**Figure 4:**
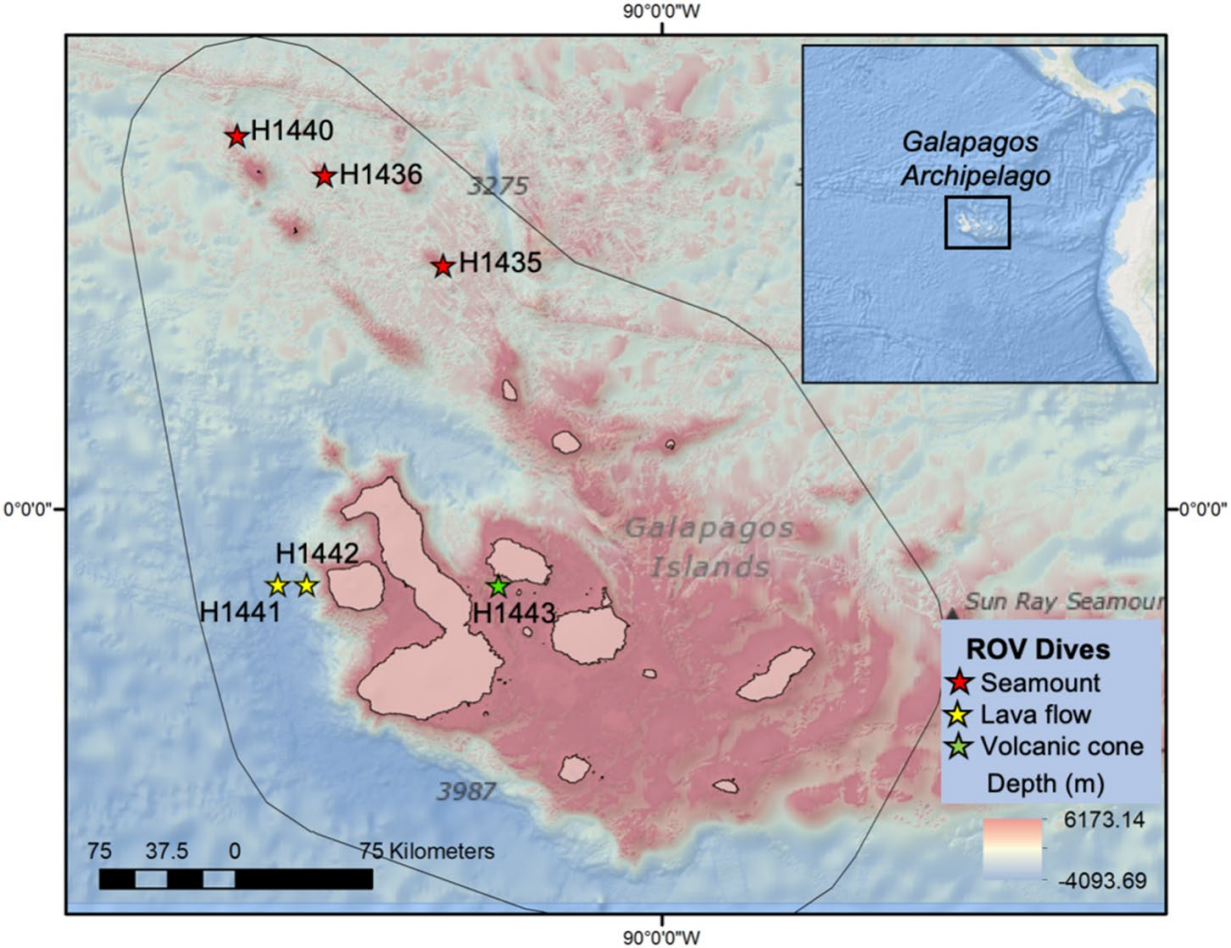
Bathymetric map of the Galapagos Marine Reserve with ROV dive locations across the Galapagos Islands during the NA064 *E/V Nautilus* cruise. All dives also fall within the BY7 Cocos Plate deep-sea benthic province as proposed by Watling et al.^49^. Gridded bathymetric data provided by the General Bathymetric Chart of the Oceans (GEBCO) 30 arc-second grid (accessed via https://www.gebco.net/). Map created in ESRI ArcMap (version 10.3.1).

From the image analysis, we present morphospecies present on young volcanic formations (0.1- < 1 Ma) around the Darwin and Wolf lineament in the north of the GMR (H1435, H1436 and H1440), and one location relatively close (< 100 km) to the Galapagos Hotspot to the west of the archipelago^47^ (H1443; Fig. 4). We only recorded major differences in morphospecies richness between shallow depth strata (200–700 m) from North and Southeast regions (Fig. 3), which could suggest the presence of different biogeographical areas within the Archipelago, as it has been proposed for shallow marine communities^39^. However, given the limited number of locations surveyed at each region, further data is required to determine whether this biogeographic division applies to deep-sea communities. In addition, given the movement of the Nazca plate east towards the South America mainland, the islands that are formed in the Galapagos hotspot travel east, subsiding to eventually become drowned islands^48^. Therefore, the islands and seamounts to the east of the archipelago represent much older geological formations, with some dated to be > 8 Ma^47^. It is thus likely that additional explorations to the east of the archipelago might result in the discovery of distinct populations and additional new taxa. Future deep-sea research directed at investigating the effect of environmental predictors on deep-sea biodiversity is also required to better understand these relationships at the Galapagos Islands.

Although deep-sea communities are rarely included into spatial-planning processes, the Ecuadorian government recently reviewed the zoning process for the Galapagos Marine Reserve and prioritized the protection of seamounts and open water environments^50^. The information presented here contributed to this spatial planning process and promoted the protection of specific areas based on the demonstration of the presence of VME indicator species and communities and unique taxa and behaviours^31,51,52^. In March 2016, a new zoning plan was proposed and fully protected areas were recommended across the reserve to protect seamounts and other deep-sea habitats, including the creation of a 40,000 km^2^ marine sanctuary to protect the three seamounts we surveyed around Darwin and Wolf islands, a hotspot for pelagic species^10,53,54^. However, due to continuous political pressure from the Galapagos artisanal fishing sector, this zoning plan is still under re-evaluation^55^.

The discovery of these diverse deep-sea biological communities highlights yet again the ecological significance of the marine ecosystems of the Galapagos Islands. This deep-sea world that Darwin never saw represents a unique and pristine environment, particularly considering the historical absence of destructive human practices known to have catastrophic impacts upon fragile communities^56,57^, such as bottom trawling or deep-sea mining; and their current conservation status and level of protection granted by the Galapagos Marine Reserve. Under the present climate crisis, that will have major effects across the world’s marine ecosystems^58^, including the deep sea^59–61^, the Galapagos Islands represent an ideal location from which to monitor future changes in this poorly studied and understood biome.

## Methods

### NA064 Galapagos platform cruise

In June 2015, we conducted a 10-day collaborative research cruise (NA064) aboard E/V *Nautilus* between the Ocean Exploration Trust, the Charles Darwin Foundation and the Galapagos National Park Directorate to explore deep-sea environments of the Galapagos Marine Reserve. All methods were carried out in accordance with relevant guidelines and regulations by the Galapagos National Park Directorate under research permits PC-26–15 & PC-45–15. All experimental protocols were reviewed and approved by a Galapagos National Park Directorate’s committee that evaluates animal care in research activities.

### ROV surveys and sample collection

Exploration of the seafloor was carried out using the two-body Remotely Operated Vehicle (ROV) system *Argus* and *Hercules*, each rated for 4 km water depth. Video and still images of the sites were acquired using Insite Pacific Zeus Plus HD color video cameras on both vehicles, each equipped with a 10 × mechanical zoom lens. Biological specimens were collected using the ROV manipulator arm and placed on sample boxes aboard *Hercules* ROV for recovery*.*

We conducted a total of six exploratory dives to the north, west, and central part of the Galapagos Archipelago (Fig. 4, Table 1). In general terms, sampling transects began at the base of each feature explored and we conducted a general upslope transect, following sonar and visual features along the way. Dives H1435, H1436 and H1440 explored three conical seamounts around the most northern islands of the archipelago, Darwin and Wolf. Dives H1441 and H1442 explored the abyssal plain located to the west of Fernandina and adjacent to the Galapagos. The last dive, H1443, explored a shallower volcanic cone, with two horseshoe-shaped craters, located between Santiago and Isabela islands in the central part of the Archipelago (Fig. 4, Table 1).

During each dive, biological samples were collected opportunistically using the ROV’s arms or suction sampler, with the aim of collecting a representative sample of specimens, but with a sampling bias towards large and conspicuous megafauna and their associates. Sampling involved the collection of individual organisms (or part of them) with their associated epibionts, as well as collecting rocks with organisms attached to them. When the ROV was back on deck, individual organisms were separated and photographed. Specimens were then sub-sampled and preserved following standardized protocols^62^ and in accordance with subsequent morphological and genetic analysis. Scleractinian corals were not included in the taxonomic analysis due to permit restrictions.

### Morphological and genetic identification of collected specimens

Whole specimens or sub-samples were sent to renowned experts on specific taxa for morphological and/or genetic identification. Most specimens were identified by morphological traits to the lowest taxonomic level. Additionally, some Annelida, Arthropoda, Mollusca and Echinodermata tissue sub-samples were sequenced for genetic characterization. For the Annelida the standard Folmer (M1-M6) region (~ 650 bp) of the mitochondrial cytochrome oxidase subunit 1 gene (mtDNA COI) was sequenced^63^. For the Echinodermata, a hybrid enrichment phylogenetic method was used to obtain sequences from 415 housekeeping genes (285 kbp)^64^.

Accepted species names were verified using WoRMS (World Register of Marine Species, 2017) and WoRDSS (World Register of Deep-sea Species, 2017). Geographical distribution of identified taxa was investigated using the OBIS (Ocean Biogeographic Information System). Voucher specimens will be stored at the Charles Darwin Foundation Galapagos Biodiversity Collection. All taxonomic identifications and metadata have been formatted to DarwinCore standard^65^.

### Presence of vulnerable marine ecosystems and imagery analysis

Due to the volume of non-quantatitive data that had been recorded from the *Hercules* ROV during the NA064 expedition, it was decided to analyse a series of still images rather than the full video sequence for each dive. Video files were converted to image sequences, with one frame extracted every 5 s using QuicktimePro 7. As the speed of the ROV varied over the duration of the full transect, an image was selected every 10 m for analysis rather than a set time interval. To achieve this, the ROV USBL navigation was cleaned and smoothed in ArcGIS (version 10.3) using editing and cartography toolboxes and split into 10 m segments. A shapefile for the mid-point of each 10 m segment was then generated. Using the join and relate functions in ArcGIS, the 5-s image frame (co-registered with the smoothed USBL positional data) that was in closest proximity to the 10 m mid-point, was selected for further analysis. Video data recorded during sampling events, were removed from the transect data. Dives H1441 and H1442 that were conducted on deep abyssal plains were excluded from the morphospecies analysis given the homogeneity of the terrain surveyed.

Image analysis was conducted using the BIIGLE 2.0 software^66^ by three analysts. To minimise bias, each analyst was randomly assigned a third of the images from each dive to analyse. To account for the variation in image quality (i.e. blue water, blurred images, variation in field of view) but to be able to obtain the maximum amount of viable data, all images were annotated using the following label trees; 1) image quality; 2) dominant and secondary substrate type; 3) presence of habitat-forming fauna (or VMEs) such as sponges, corals or bivalves; or large aggregations of benthic fauna such as brittlestars or featherstars within the field of view of the oblique high-definition camera and finally; 4) the presence of morphospecies (> 3 cm in size) identified to the lowest taxonomic level possible. Only images of sufficient quality were analysed. For the purpose of this study VME communities were defined as follows; 1) coral gardens were defined as the presence of > 5 colonies of octocorals and/or hexacorals (not including scleractinians) of the same or different morphospecies; 2) sponge gardens were defined as the presence of > 5 colonies of erect (not encrusting) of the same or different morphospecies and; 3) cold-water coral communities were defined as the presence of 3D habitat-forming scleractinian colonies. Scaling lasers set 10 cm were used to opportunistically to measure biota or geological formations. However, as these were not used consistently for the duration of the dive and owing to the oblique camera angle, densities of morphospecies have not been calculated. To ensure consistency between analysts, any conflicts in morphospecies identification or VME community identification were resolved using the “largo” re-evaluation function in BIIGLE. A total of 2077 individual images were analysed across the five ROV dives.

A species matrix was created by enumerating taxonomically distinct organisms in each image as present or absent over each 100 m depth band. Given the uncertainties of identifying morphospecies from ROV imagery we adopted best practices in analyses to provide the best possible yet conservative identifications, and where appropriate, morphospecies have been grouped at higher taxonomic levels where identifications were uncertain. Indeterminate taxa that could not be confidently distinguished or identified to phylum were excluded from the analyses.

## Supplementary information

Supplementary information

## Data Availability

Data cannot be shared publicly because of Galapagos National Park research permit conditions. A previous authorization from the Galapagos National Park Directorate is required for further use of this data. Access to data can be requested via this email address at the Galapagos National Park Directorate Applied Research Department: investigacion@galapagos.gob.ec.
