# Supplementary material for: Characterization of deep-sea benthic invertebrate megafauna of the Galapagos Islands
Source: Sci Rep. 2020 Aug 17;10:13894. doi: 10.1038/s41598-020-70744-1 (PMC7431423; doi:10.1038/s41598-020-70744-1)

**Supplementary materials**

**Characterization of deep-sea benthic invertebrate megafauna of the Galapagos Islands.**

Pelayo Salinas-de-León^1,2,*^, Patricia Martí-Puig^1^, Salome Buglass^1^, Camila Arnés-Urgellés^1^, Etienne Rastoin^1^, Marie Creemers^1^, Stephen Cairns^3^, Charles Fisher^4^, Timothy O’Hara^5^, Bruce Ott^6^, Nicole A. Raineault^7^, Henry Reiswig^8^, Greg Rouse^9^, Sonia Rowley^10^, Timothy M. Shank^11^, Jenifer Suarez^12^, Les Watling^13^, Mary K. Wicksten^14^, Leigh Marsh^1,15^


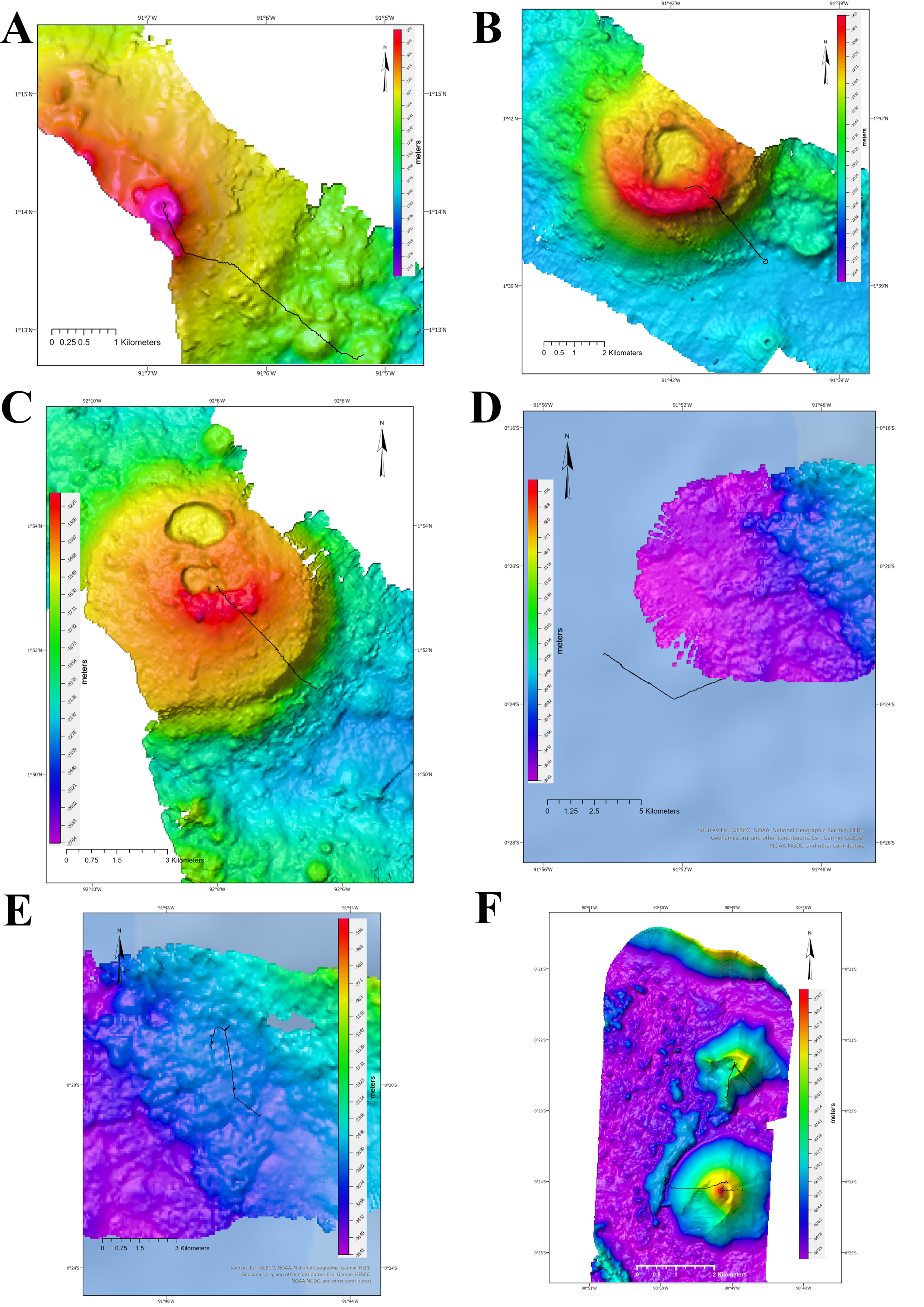


**Supplementary material 1.** ROV dives from the NA064 Expedition. Ship-acquired multibeam with ROV USBL navigation. Gridded background bathymetric data provided by the General Bathymetric Chart of the Oceans (GEBCO) 30 arc-second grid (accessed via <http://www.gebco.net/>). Map created in ESRI ArcMap (version 10.3.

**Supplementary material 2.** List of morphospecies recorded on ROV video transects collected on seamounts (H1435, H1436, H1440) and volcanic cones (H1443A, H1443B) of the Galapagos Marine Reserve.

| **Phylum** | **Class** | **Order** | **Lowest taxonomic rank** | **Morphotype or morphospecies** | **H1435** | **H1436** | **H1440** | **H1443A** | **H1443B** |
| --- | --- | --- | --- | --- | --- | --- | --- | --- | --- |
| Annelida | Polychaeta | Terebellida | Genus | *Biremis* sp. indet. | x | x | x | - | - |
| Arthropoda | Malacostraca | Decapoda | Infraorder | Anomura spp. stet. | x | x | x | x | x |
|  |  |  | Family | Chirostylidae spp. stet. | x | x | - | - | x |
|  |  |  | Genus | *Eumnida* sp. indet. | x | - | - | x | x |
|  |  |  | Family | Sternostylidae spp. indet. | x | x | - | x | x |
|  |  |  | Family | Cancridae sp. indet. | - | - | - | x | x |
|  |  |  | Family | Homolidae sp. indet. | x | - | - | - | - |
|  |  |  | Family | Lithodidae spp. indet. | x | - | - | - | - |
|  |  |  | Family | Epialtidae sp. indet. | x | - | - | - | - |
|  |  |  | Family | Mathildellidae sp. indet. | x | - | - | - | - |
| Chordata | Thalicea | Pyrosomatida | Family | Pyrosomatidae sp. indet. | x | - | - | - | - |
|  | Ascidiacea | Phlebobranchia | Family | Octacnemidae sp. indet. | - | - | x | - | - |
| Cnidaria | Anthozoa | Actiniaria | Order | Actiniaria spp. indet. | x | x | x | x | x |
|  |  |  | Order | Actiniaria sp. indet. | x | - | - | x | x |
|  |  |  | Family | Actinoscyphiidae spp. indet. | x | x | - | - | - |
|  |  | Antipatharia | Genus | *Stichopathes* sp. indet.* | - | - | x | x | x |
|  |  |  | Morphotype | Antipatharia (3D branching) spp. indet.* | x | x | x | x | x |
|  |  |  | Morphotype | Schizopathidae spp. indet.* | x | x | x | x | x |
|  |  | Corallimorpharia | Family | Corallimorphidae sp. indet. | x | - | - | - | - |
|  |  | Scleractinia | Morphotype | Scleractinia (cup coral type) spp. indet. | x | x | - | x | - |
|  |  |  | Species | *Dendrophyllia californica** | x | - | - | x | x |
|  |  |  | Species | *Madrepora oculata** | x | - | - | x | - |
|  |  |  | Morphotype | Scleractinia (branching type) spp. indet.* | x | - | - | x | x |
|  |  | Zoantharia | Family | Parazoanthidae sp. stet. | x | x | x | - | - |
|  |  | Alcyonacea | Family | Acanthogorgiidae sp. indet.* | x | x | x | x | x |
|  |  |  | Suborder | Alcyoniina sp. stet.* | x | - | - | x | - |
|  |  |  | Family | Alcyoniidae spp. indet.* | x | x | x | x | x |
|  |  |  | Family | Clavulariidae sp. indet.* | x | - | - | x | - |
|  |  |  | Family | Chrysogorgiidae spp. indet.* | x | x | x | x | x |
|  |  |  | Family | Isididae spp. indet.* | x | x | x | x | x |
|  |  |  | Family | Paragorgiidae sp. indet.* | - | x | - | - | - |
|  |  |  | Family | Plexauridae sp. indet.* | x | x | x | x | x |
|  |  |  | Family | Primnoidae spp. indet.* | x | x | x | x | x |
|  |  |  | Genus | *Victorgorgia* sp. indet.* | x | x | - | - | - |
|  |  |  | Morphotype | Anthozoa (whip-like) spp. stet.* | - | - | x | x | x |
|  |  | Pennatulacea | Genus | *Anthoptilum* sp. indet.* | x | - | - | - | - |
|  |  |  | Family | Halipteridae sp. indet.* | x | - | x | - | - |
|  |  |  | Family | Pennatulidae sp. indet.* | x | x | x | - | - |
|  |  |  | Family | Umbellulidae sp. indet.* | - | x | - | - | - |
|  | Hydrozoa | Anthoathecata | Family | Corymorphidae sp. stet | - | - | x | - | - |
|  |  |  | Genus | *Crypthelia* sp. indet.* | x | x | x | x | x |
|  |  | Siphonophora | Order | Siphonophora sp. stet | - | - | - | x | - |
| Ctenophora |  |  | Phylum | Ctenophora sp. stet | - | - | - | x | - |
| Echinodermata | Asteroidea |  | Class | Asteroidea spp. stet. | x | x | x | x | x |
|  |  | Brisingida | Order | Brisingida spp. indet. | x | x | x | x | x |
|  |  |  | Superorder | Valvatacea spp. indet. | x | x | x | x | x |
|  | Crinoidea | Comatulida | Order | Comatulida spp. indet. | x | x | x | x | x |
|  |  |  | Morphotype | Stalked crinoids spp. indet. | x | - | - | x | - |
|  | Echinoidea | Echinothurioida | Order | Echinothurioida sp. stet. | x | x | x | - | - |
|  |  | Cidaroida | Genus | *Histocidaris* sp. indet. | x | x | - | x | x |
|  |  |  | Species | *Centrocidaris doederleini* | x | x | x | x | x |
|  | Holothuroidea |  | Class | Holothuroidea spp. stet. | x | x | x | x | x |
|  |  | Elasipodida | Family | Elpidiidae spp. indet. | - | x | x | - | - |
|  |  | Synallactida | Family | Synallactidae sp. indet. | - | x | x | x | x |
|  | Ophiuroidea | Euryalida | Family | Euryalidae sp. indet. | x | x | x | - | - |
|  |  |  | Family | Gorgonocephalidae sp. indet. | - | - | - | - | x |
|  |  | Ophiuroidea | Infraclass | Metophiurida spp. indet. | x | x | x | x | - |
| Foraminifera | Monothalamea |  | Superfamily | Xenophyophoroidea spp. indet.* | x | x | x | - | - |
| Mollusca | Bivalvia | Limida | Family | Limidae sp. indet. | x | - | - | - | - |
|  |  | Pectinida | Family | Pectinidae sp. indet. | x | - | - | - | - |
| Porifera | Hexactinellida | Sceptrulophora | Order | Sceptrulophora spp. indet.* | x | x | x | - | - |
|  |  | Lyssacinosida | Order | Lyssacinosida spp. indet.* | x | x | x | - | x |
|  |  |  | Genus | Regadrella sp. indet.* | x | x | x | x | x |
|  |  |  | Family | Rossellidae spp. indet.* | x | - | - | x | - |
|  |  |  | Family | Bolosominae spp. indet.* | - | - | x | - | - |
|  |  |  | Genus | *Saccocalyx* sp. indet.* | - | x | - | - | - |
|  |  | Amphidiscosida | Family | Hyalonematidae sp. indet.* | x | x | x | - | x |
|  | Demospongiae | Poecilosclerida | Family | Cladorhizidae (stalk) sp. indet.* | x | x | x | - | - |
|  |  |  | Family | Cladorhizidae (bushy) sp. indet.* | x | - | - | - | - |
|  |  | Polymastiida | Order | Penicillaria sp. stet. | x | x | - | x | - |

**Supplementary material 3.** Bathymetric variation on oxygen concentration from ROV mounted CTD and Oxygen optode. The depth distribution of the Oxygen Minimum Zone (OMZ) is highlighted in red.


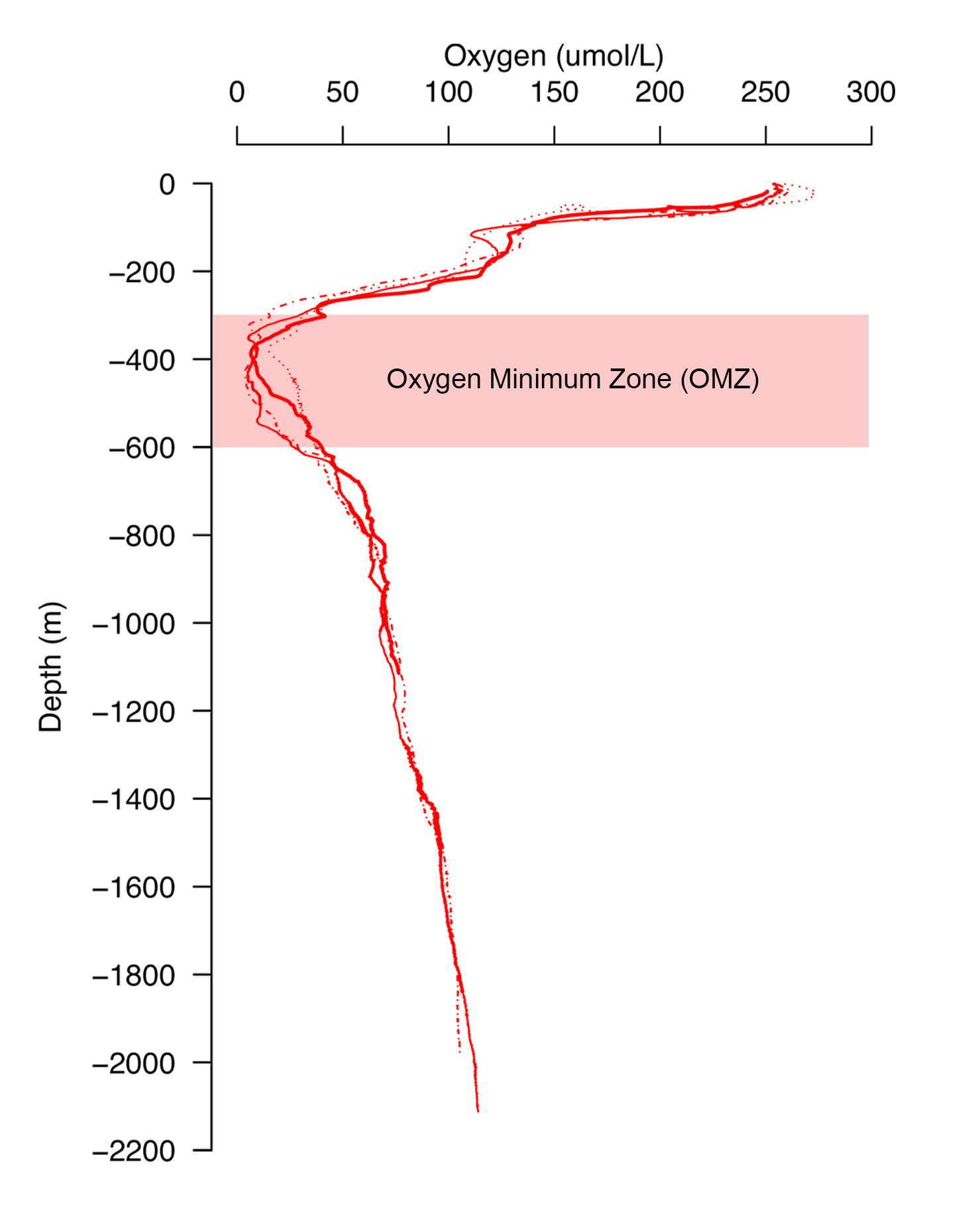

Supplement: Supplementary file 1 — Supplementary information [file 41598_2020_70744_MOESM1_ESM.docx]
